# Editorial: Functional and smart biomaterials: Development and application in regenerative medicine-Volume II

**DOI:** 10.3389/fbioe.2022.1120438

**Published:** 2023-01-04

**Authors:** Guicai Li

**Affiliations:** Co-innovation Center of Neuroregeneration, Key laboratory of Neuroregeneration of Jiangsu and Ministry of Education, NMPA Key Lab for Research and Evaluation of Tissue Engineering Technology Products, Nantong University, Nantong, China

**Keywords:** functional biomaterials, smart biomaterials, development, application, regenerative medicine

Functional and smart biomaterials are biomaterials that have controllable properties and can perform beneficial treatment and repair effect in human body (Kowalski et al., 2018; Khan et al., 2022). Due to their attractive biological properties and smart responsiveness, they show promising potential in various regenerative medicine fields ranging from wound healing applications to tissue regeneration and reconstruction, etc (Li et al., 2020; Najjari et al., 2022). This Research Topic covers a Research Topic of articles on Functional and Smart Biomaterials: Development and Application in Regenerative Medicine-Volume II, based on the first Special Volume of this Research Topic.

In this Research Topic, we have collected a total of seven papers, which cover the preparation, characterization, evaluation and current research progress of the functional and smart biomaterials in various biomedical fields. We thus have briefly introduced the papers published in the Research Topic as follow.

The first article of this Research Topic by Xie, et al. introduced the preparation of wood patches coated with rhodamine and rapamycin, and evaluated their effect by implanting into the rat subcutaneous tissue, the abdominal cavity, or the inferior vena cava (IVC). Wood patches became soft after processing with Na_2_SO_3_ (>98%) and NaOH (>98%), and showed biocompatibility after implantation into the subcutaneous tissue or the abdominal cavity. The natural microstructure in the wood patch facilitated attachment and migration of neointimal endothelial progenitor cell, as well as therapeutic drug loading and continuous release *in vivo*. The study showed that the wood-derived scaffolds could be used successfully as vascular patches and a drug delivery system, suggesting the potential application of plant-derived materials in vascular applications.

Hydrogel-based tissue engineering has been widely used to repair cartilage injury. However, whether this approach can be applied to treat nasal septum cartilage defects remains unclear. In the study by Zhang, et al., three gelatin methacrylate-based scaffolds loaded with transforming growth factor (TGF)-β1 (GelMA-T) were prepared, and their repair effect on nasal septum cartilage defects was examined. They proved that at 4, 12, and 24 weeks post surgery, the nasal septum cartilage defects exhibited more complete repair in rabbits treated with the 10% GelMA-T/BMSC scaffold according to hematoxylin and eosin, safranine-O, and toluidine blue staining, which could be applied for the physiological and structural repair of defects in the nasal septum cartilage, thus providing a potential strategy for repairing cartilage defects in the clinic.

Hyaluronic acid (HA) and its derivatives have played an increasingly important role in tissue engineering, wound healing, cancer treatment, ophthalmology, and cosmetics. Yasin, et al.highlighted and summarized the recent progress in converting HA to smart formulation, such as multifunctional coatings, targeted nanoparticles, or injectable hydrogels, which are used in advanced biomedical application. The relationship between the molecular properties of HA and the advances in biomedical applications was systematically explored. The future application of HA on composite biomedical platform systems was summarized. This review provided a comprehensive knowledge relevant to the development of the composite biomedical platform systems of HA.

In another study, Shang, et al. systematically evaluated the corrosion behaviors of Mg-based vascular stent materials include pure Mg, AZ31, and WE43 in a dynamic environment and studied the effect of their degradation products on the behavior of vascular cells. The results showed that the corrosion rate of different Mg based materials was related to the composition of the elements. The effect of corrosive products on vascular cells was beneficial to re-endothelialization and inhibition of smooth muscle cell proliferation at the implantation site of vascular stent materials, which may have potential application as a potential vascular stent material.

Yue, et al. analyzed and summarized existing biomaterial delivery and restoring platforms, and the therapeutic strategies based on biomaterial platforms, including general strategies to block the fibrosis process and new strategies to promote cellular restoring effects. They proposed that the development of structures with the ability to block further fibrosis progression as well as to promote cardiomyocytes viability should be the main research interests in myocardial fibrosis, and the reestablishment of structures necessary for normal cardiac function was central to the treatment of myocardial fibrosis. Further on, the future application of biomaterials for myocardial fibrosis was also conducted.

Non-traumatic osteonecrosis of the femoral head (NONFH) remains a common refractory disease with poorly understood pathogenesis. Tan et al. described direct visual evidence for the involvement of dynamic changes in macrophages and the chronic inflammatory microenvironment in human NONFH. They proved that macrophage M1/M2 imbalance facilitated the progression of NONFH, osteonecrosis and tissue fibrosis in the local lesion. Inhibiting inflammation, promoting the resolution of inflammation, switching macrophages to an M2 phenotype, or inhibiting their adoption of an M1 phenotype, which may be useful therapeutic strategies against NONFH in future.

Xu et al. constructed a radiomics model to predict cognitive dysfunction in patients with Type 2 diabetes mellitus (T2DM) in terms of MRI-based radiomics features. Compared with the Support Vector Machine (SVM) and k-NearestNeighbor (KNN), the Logistic Regression (LR) algorithm for the construction of the model showed better performance. Thus, the model may serve as a potential tool to guide personalized treatment.

In summary, the functional and smart biomaterials have been broadly studied in various regenerative medicines, including wound treatment, tissue culture, targeted therapy of diseases, and tissue regeneration, *etc.* Here, seven top quality articles have been published in this collected Research Topic on Functional and Smart Biomaterials: Development and Application in Regenerative Medicine-Volume II. In prospectively, we hope this Research Topic would be meaning for development and application of novel functional and smart biomaterials, such as the design, the evolution of advanced manufacture techniques, and biomedical applications in the field of regenerative medicine and tissue engineering.

## References

[B1] Khan H. M. Liao X. Sheikh B. A. Wang Y. Su Z. Guo C. (2022). Smart biomaterials and their potential applications in tissue engineering. J. Mater Chem. B 10 (36), 6859–6895. 10.1039/d2tb01106a 36069198

[B2] Kowalski P. S. Bhattacharya C. Afewerki S. Langer R. (2018). Smart biomaterials: Recent advances and future directions. ACS Biomater. Sci. Eng. 4 (11), 3809–3817. 10.1021/acsbiomaterials.8b00889 33429614

[B3] Li Z. Mei S. Dong Y. She F. Li Y. Li P. (2020). Functional nanofibrous biomaterials of tailored structures for drug delivery—a critical review. Drug Delivery-A Crit. Rev. Pharm. 12 (6), 522. 10.3390/pharmaceutics12060522 PMC7355603 32521627

[B4] Najjari A. Mehdinavaz Aghdam R. Ebrahimi S. A. S. Suresh K. S. Krishnan S. Shanthi C. (2022). Smart piezoelectric biomaterials for tissue engineering and regenerative medicine: A review. Biomed. Tech. Berl. 67 (2), 71–88. 10.1515/bmt-2021-0265 35313098

